# Characteristics of Isolates of *Pseudomonas aeruginosa* and *Serratia marcescens* Associated With Post-harvest Fuzi (*Aconitum carmichaelii*) Rot and Their Novel Loop-Mediated Isothermal Amplification Detection Methods

**DOI:** 10.3389/fmicb.2021.705329

**Published:** 2021-08-20

**Authors:** Meng Fu, Xin Zhang, Bei Chen, Mingzhu Li, Guoyan Zhang, Langjun Cui

**Affiliations:** ^1^The Key Laboratory of Medicinal Resources and Natural Pharmaceutical Chemistry, National Engineering Laboratory for Resource Development of Endangered Crude Drugs in Northwest China, The Ministry of Education, College of Life Sciences, Shaanxi Normal University, Xi’an, China; ^2^Chenggu County Qunli Traditional Chinese Medicine Cooperative, Chenggu, China

**Keywords:** *Aconitum carmichaelii* Debx., post-harvest, rot, *Pseudomonas aeruginosa*, *Serratia marcescens*, loop-mediated isothermal amplification

## Abstract

Fuzi (the lateral root of *Aconitum carmichaelii* Debx.) is a traditional Chinese medicine that is cultivated in more than eight provinces in China. However, it can be easily devastated by post-harvest rot, causing huge losses. Therefore, it is extremely important that the primary causal pathogens of post-harvest Fuzi rot are identified and appropriate detection methods for them are developed to prevent and control losses. In this study, two bacterial strains (X1 and X2) were isolated from rotten post-harvest Fuzi. Based on their morphological, physiological, and biochemical characteristics, housekeeping gene homologies, and matrix-assisted laser desorption ionization/time-of-flight mass spectrometry (MALDI-TOF MS) results, these isolates were identified as *Pseudomonas aeruginosa* and *Serratia marcescens.* The pathogenicities of these isolates were confirmed by fulfilling Koch’s postulates demonstrating that they were post-harvest Fuzi rot pathogens. Two loop-mediated isothermal amplification (LAMP) methods targeting the gyrase B subunit (*gyrB*) gene of *P. aeruginosa* and the phosphatidylinositol glycan C (*pigC*) gene of *S. marcescens* were successfully developed, and it was found that the target genes were highly specific to the two pathogens. These LAMP methods were used to detect *P. aeruginosa* and *S. marcescens* in 46 naturally occurring Fuzi and their associated rhizosphere soil samples of unknown etiology. The two bacterial assays were positive in some healthy and rotten samples and could be accomplished within 1 h at 65°C without the need for complicated, expensive instruments. To our knowledge, this is the first report of *P. aeruginosa* and *S. marcescens* causing post-harvest Fuzi rot. The newly developed methods are expected to have applications in point-of-care testing for the two pathogens under different Fuzi planting procedures and will significantly contribute to the control and prevention of Fuzi rot.

## Introduction

Post-harvest diseases of crops are a significant issue worldwide, causing the loss of approximately 20–40% of global agricultural production (Roberto et al., 2019). Consequently, post-harvest processing, advanced disease detection, and disease prevention are extremely to minimize disease-induced damage in crops (Fang and Ramasamy, 2015). The most important post-harvest pathogens of most fruits, vegetables, and crops have now been identified and are detected using laboratory-based techniques including polymerase chain reaction (PCR), quantitative real-time PCR (qRT-PCR), immunofluorescence (IF), fluorescence *in situ* hybridization (FISH), enzyme-linked immunosorbent assay (ELISA), and loop-mediated isothermal amplification (LAMP) (Fang and Ramasamy, 2015). Each method has its advantages and limitations for disease detection, with powerful, inexpensive, and convenient methods being preferable.

**FIGURE 1:**
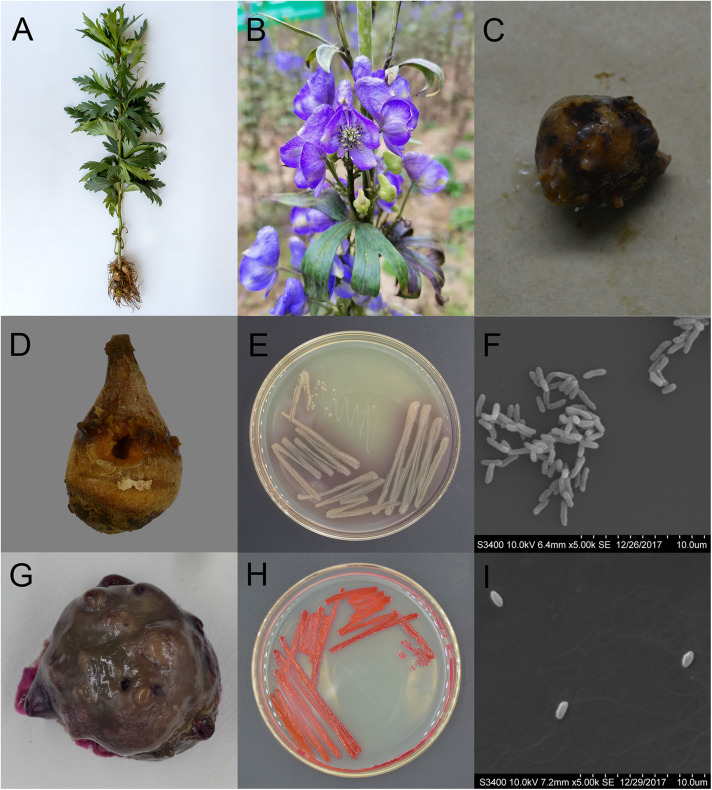
Characteristics of *Aconitum carmichaelii* and the two isolated pathogens, Pseudomonas aeruginosa and Serratia marcescens. **(A)** *Aconitum carmichaelii* cultivars. **(B)** *Aconitum carmichaelii* flower. **(C)** Rotten Fuzi under natural conditions. **(D)** Rotten Fuzi inoculated with *Pseudomonas aeruginosa* X1. **(E)** The morphology of *Pseudomonas aeruginosa* X1 colonies on KB agar medium. **(F)** Cell of *Pseudomonas aeruginosa* X1 as observed by SEM at a magnification of ×5,000. **(G)** Rotten Fuzi inoculated with *Serratia marcescens* X2. **(H)** The morphology of *Serratia marcescens* X2 colonies on LB agar medium. **(I)** Cell of *Serratia marcescens* X2 as observed by SEM at a magnification of ×5,000.

*Aconitum carmichaelii* Debx. is a traditional Chinese medicinal plant (Figures 1A,B), the lateral root of which (known as Fuzi in Chinese) has been widely applied in Asia as an essential herbal drug for 2000 years. Fuzi has been used to treat various diseases, such as rheumatism, cardiovascular diseases, painful joints, syncope, and bronchial asthma (Xia et al., 2021). In the main planting regions in China, people also eat heat-cooked Fuzi in winter to prevent colds (Kao and Zhang, 2013). Due to its excellent therapeutic effects, there is a great demand for Fuzi in traditional Chinese medicine, with *A. carmichaeli* currently being cultivated in more than eight provinces in China, and the artificial planting regions having rapidly expanded in recent years. In the main planting regions of Hanzhong (Shaanxi Province) and Jiangyou (Sichuan Province), *A. carmichaelii* is often planted in the first November and harvested the following summer (from late June to early August). Under high temperature and humid conditions, post-harvest Fuzi is quickly decays within 12 h (Figure 1C), so harvested Fuzi is usually soaked in 20–30% Danba solution (main ingredient: CaCl_2_) to prevent decay (Chen et al., 2020). However, this procedure can result in the various Fuzi processed products having high Danba residues, which further affects safe medication practices (Chen et al., 2020). Although low-temperature storage could prevent decay, it could increase the cost of the herb. Therefore, screening the available methods to prevent post-harvest Fuzi decay could minimize losses. However, the primary pathogens that cause post-harvest Fuzi decay and the appropriate detection methods for them are currently unknown.

In this study, *Pseudomonas aeruginosa* and *Serratia marcescens* were identified as causal pathogens of post-harvest Fuzi rot and novel LAMP methods were developed for their detection. It is hoped that these powerful methods will be convenient tools for detecting the two post-harvest Fuzi rot pathogen species, allowing companies to make more informed and timely decisions regarding their management.

## Materials and Methods

### Isolation and Identification of the Post-harvest Pathogens

In July 2016, rotten and healthy post-harvest Fuzi materials were collected from Mianyang (Sichuan Province), Lijiang (Yunnan Province), and Hanzhong (Shaanxi Province) in China. Diseased tissue was excised from the rotten root and disinfected in 70% ethanol solution for 20 s, soaked in 0.1% NaOCl for 50 s, and rinsed in sterilized water three times. They were then cut with a sterilized blade, and a small block was taken from the inside of each sample and cultured on Luria-Bertani (LB) agar at 37°C. This resulted in 10 bacterial strains being isolated which were further cultured with LB liquid medium. Surface-sterilized healthy Fuzi were each inoculated with one of the isolate suspensions [10^8^ colony-forming units (CFU)/mL] or distilled and deionized water (ddH_2_O) as a blank control (*n* = 15 Fuzi per treatment consisting of three replicates). The inoculated Fuzi were then placed in a plant growth chamber (30°C and 70% relative humidity) for 15 days, and any apparent symptoms were recorded. When the visible rot zone beyond the wounded area on each Fuzi surface was more than 2 mm wide, it was scored as infected. The incidence of disease was calculated and expressed as a percentage (%), using the formula:

Incidenceofdisease(%)=NumberofinfectedFuzi/totalnumberofFuzi×100%.

It was found that only 2 of the 10 candidate bacterial isolates caused Fuzi rot. Therefore, these two strains were named X1 and X2. Then the two bacterial strains were re-isolated from the rotten root materials, and further confirmed based on the following detailed morphological and physiological analyses.

The morphological characteristics of isolates X1 and X2 were assessed using scanning electron microscopy, while their physiological and biochemical characteristics were analyzed according to Brenner et al. (2004) and the VITEK 2 COMPACT system (bioMérieux, Marcy-l’Étoile, France) based on Clinical and Laboratory Standards Institute guidelines.

Genomic DNA was extracted from isolates X1 and X2 using a bacterial genomic DNA extraction kit (Takara Bio Inc., Shiga, Japan) following the manufacturer’s instructions. The 16S rRNA gene was then amplified using the universal bacterial primer pair 27F and 1492R (Table 1). The reaction system contained 12.5 μL of 2 × Taq PCR (Takara Bio Inc., Shiga, Japan), 1 μL of the F primer, 1 μL of the R primer, 20 ng of the DNA template, and enough ddH_2_O to make a final volume of 20 μL. The PCR products were separated by agarose gel electrophoresis and purified with a commercial kit (Axygen, Union City, United States). The purified products were then sequenced by Sangon Biotech (Shanghai) Co., Ltd., and the sequences were aligned with the Nucleotide Basic Local Alignment Search Tool (BLASTN) online database. The housekeeping genes gyrase B subunit (*gyrB*) of X1 (Savli et al., 2003) and phosphatidylinositol glycan C (*pigC*) of X2 (Gillis et al., 2014) were amplified with relevant primers (Table 1) and sequenced. The 16S rRNA gene sequences and housekeeping gene sequences of closely related bacteria were then downloaded from the National Center for Biotechnology Information (NCBI),^1^ and a phylogenetic tree was constructed using the neighbor-joining method and assembled in the MEGA 6.0 program.

**TABLE 1:** Primer sets used for polymerase chain reaction (PCR) and the loop-mediated isothermal amplification (LAMP) assays.

	Name	Primers sequences	Annealing temperature
PCR primer	16S rDNA-27F	AGAGTTTGATCCTGGCTCAG	53°C
	16S rDNA-1492R	GGTTACCTTGTTACGACTT	
	*gyrB*-1F	CGACAATGGACGCGGTAT	55°C
	*gyrB*-1R	CCTTGGCTTCGTTGGGATT	
	*pigC*-F	AGCTGGAGCGGGAACTCA	58°C
	*pigC*-R	GCCGTCGAGAATCAAGGTGT	
LAMP primer	*gyrB*	**F3:** CAAGGTGTCCGGCGGCTT; **B3:** GGCTTGAAGTGAACTT CGGT; **FIP:** TTGTGGCGACGGATGGTCAG-CGGTGTGGGC GTCTCGG; **BIP:** GGTCTGGGAACAGGTCTACC-ATCGGTC TCGCCCACTTC; **LF:** TAGTTCATGGGACAGCGCGT; **LB:** GGC GTTCCGCAGTTCCCA;	Constant 65°C
	*pigC*	**F3:** GCGTTGAAGGCCCTGTTC; **B3:** GCAGGTAGCTGGGATC GTC; **FIP:** CAGAAGGCGCGCGATTCGG-GAGCGACACAGCG CACA; **BIP:** CAAAGCGACTTCAGCGCCTTC-CCAGCGCGGAA GACTCAA; **LF:**GGTGGGCAGGACGGTGAC; **LB:** TTCGAGTTCGGCGCCCGT	Constant 65°C

The identifications of the two isolates were further confirmed by VITEK^®^ mass spectrometry (MS) based on matrix-assisted laser desorption ionization/time-of-flight (MALDI-TOF) technology (bioMérieux, Marcy-l’Étoile, France) following the manufacturer’s recommendations. One colony of each isolate was directly spotted on the manufacturer’s proprietary sample plates following the manufacturer’s protocols and recommendations. α-Cyano-4-hydroxycinnamic acid matrix solution (1 μL; bioMérieux, Marcy-l’Étoile, France) was then applied to each sample and air-dried for 5 min at room temperature for crystallization. A total of three spots were analyzed on the VITEK MS system to identify the species of each isolate.

### LAMP and PCR Primer Design and Reaction Systems

The specific LAMP primer sets of X1 and X2 were designed to target the housekeeping genes of *gyrB* and *pigC* using primer Explorer V5^2^ and included two inner primers [forward inner primer (FIP) and backward inner primer (BIP)], two outer primers (forward primer F3 and backward primer B3), and two loop primers [loop forward primer (LF) and loop backward primer (LB)] (Supplementary Figure 1 and Table 1). The PCR amplification primers were designed using the primer 5 software (Table 1). All primers were synthesized by Sangon Biotech (Shanghai) Co., Ltd., LAMP reactions were conducted according to Zhao et al. (2010). The optimized LAMP reaction mixture (25 μL) consisted of 1.5 × GelGreen^®^ (Biomed, Beijing, China), 1 × ThermoPol^®^ Reaction Buffer (New England Biolabs, Ipswich, United States), 8 mmol/L of MgSO_4_, 1 mol/L of betaine (Sigma-Aldrich, St. Louis, United States), 1.6 mmol/L of each deoxynucleotide (dNTP), 0.2 μmol/L of each outer primer (F3 and B3), 0.4 μmol/L of each LF and LB primer, 0.8 μmol/L of each inner primer (FIP and BIP), 8 U of Bst DNA polymerase (New England Biolabs, Ipswich, United States), and 5 μL of DNA template. The LAMP reaction mixture was heated at 65°C for 60 min and the reaction was terminated by heating at 85°C for 5 min. The color of the solution, which was observed with the naked eye under blue light (470 nm), turned bright green in the presence of LAMP products and remained dark red in the absence of amplification. PCR reactions were conducted as described above. In addition, 3 μL of the products of both the LAMP reaction and PCR amplification were further detected on a standard 1% agarose gel.

### LAMP Specificity and Sensitivity Tests

The two isolates X1 and X2, and the other closely related bacterial strains (Supplementary Table 1) were cultured with LB liquid medium. Their DNAs were then extracted and further used to test primer specificity. The DNAs of X1 and X2 were diluted to 20 ng/μL, 2 ng/μL, 200 pg/μL, 20 pg/μL, 2 pg/μL, 200 fg/μL, and 20 fg/μL, respectively, and used as templates for sensitivity testing. DNA extraction, PCR amplification, and LAMP were then carried out as described above.

### Application of the LAMP Assays

A total of 46 healthy and rotten post-harvest Fuzi, and their associated rhizosphere soil samples were collected in Mianyang (Sichuan Province), Lijiang (Yunnan Province), and Hanzhong (Shaanxi Province) (Supplementary Table 3). Both X1 and X2 were isolated and identified in sterilized Fuzi according to the above methods and in the rhizosphere soil samples using standard procedures. In addition, both sterilized Fuzi and soil DNAs were extracted with a commercial plant and soil genomic extraction kit (Tiangen, Beijing, China) and the two bacteria were detected in these samples using LAMP and PCR methods, as described above.

### Statistical Analysis

All of the physiological and biochemical analyses, LAMP method establishment procedures, and sample assays were repeated at least four times. The data were analyzed through the chi-square test to determine whether the observations meet the theoretical expectations. The significance level when calculating the chi-square value is set to 0.05.

## Results

### Identification and Characterization of Isolate X1

After 15 days inoculation, Fuzi inoculated with both ddH_2_O and the 8 candidate bacterial isolates showed no rotten apparent symptoms. However, Fuzi inoculated with the two isolates X1 and X2 showed visible rotten symptoms, and the incidences of disease were 100% which were different significantly with the control groups [X^2^ = 15, X^2^_(df = 1, α = 0.05)_ = 3.84]. The Fuzi that were inoculated with isolate X1 showed symptoms that were identical to those observed in rotten post-harvest Fuzi, with the appearance of yellow lesions that were sunken and contained mucus (Figure 1D). Isolate X1 could grow well on King’s B (KB) medium, with the individual colonies being smooth, round, and yellowish-green or blue-green on KB medium (Figure 1E), and the individual cells being short, straight rods with dimensions of approximately 0.5–1.0 × 1.5–3.0 μm (Figure 1F). This bacterium could hydrolyze gelatin but not starch and could utilize mannitol but not maltose, xylose, γ-aminobutyrate, or ethylene glycol. The bacterium could also grow at temperatures up to 42°C but not at 46 or 4°C. Moreover, it could not grow in KB medium containing 8.5% NaCl. Analysis by the VITEK 2 COMPACT system indicated that L-malate, D-glucose, D-trehalose, xylitol, L-arabinose, acetate, and D-gluconate were assimilated, while erythritol, D-cellobiose, L-rhamnose, D-sorbitol, and sucrose were not assimilated by this bacterium.

**FIGURE 2:**
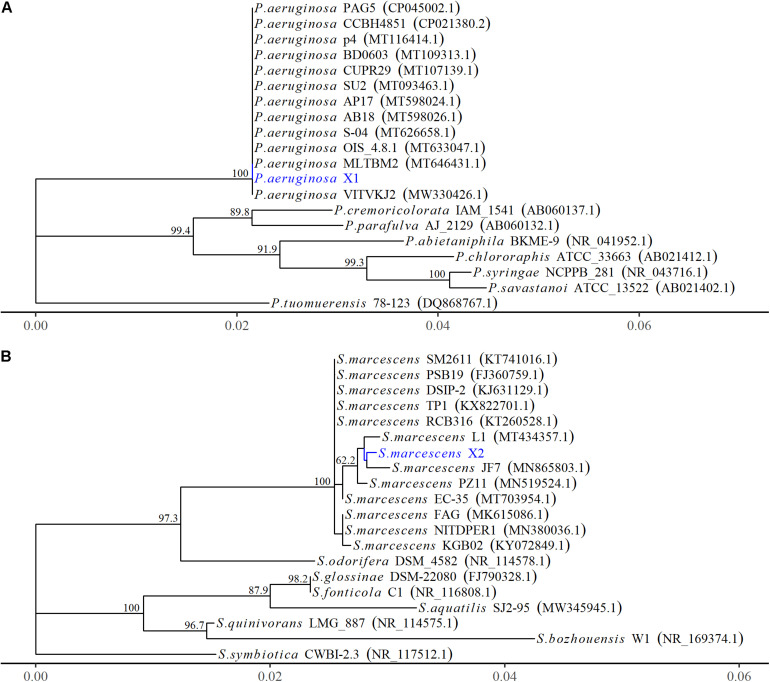
The phylogenetic relationships of *Pseudomonas aeruginosa* and *Serratia marcescens* strains based on 16S rRNA gene. **(A)** The phylogenetic tree of *Pseudomonas aeruginosa* X1. **(B)** The phylogenetic tree of *Serratia marcescens* X2. The Phylogenetic tree was constructed using MEGA-X based on the maximum likelihood method, using the Kimura 2-parameter model. All bootstrap values > 50 from 1,000 replications are shown on the interior branch nodes. The sequences obtained in this study are in blue. GenBank accession number is provided next to the tick species name.

The 16S rRNA gene of X1 was 1,378 bp in length (MW652657.1) and shared 100% identity with the other *P. aeruginosa* strains (MW.330426.1 and MT633047.1) (Figure 2A). Moreover, the housekeeping gene *gyrB* of X1 (MW691198) shared 100% identity with *P. aeruginosa* CP030910.1 and JUVT01000188.1. The MALDI-TOF MS results indicated that X1 was *P. aeruginosa* (confidence > 99.0%) (Supplementary Figure 2A and Supplementary Table 2). Therefore, based on the morphological and physiological observations, the phylogenetic analysis, and MALDI-TOF MS results, isolate X1 was classified as *P. aeruginosa*. Moreover, after X1 was reinoculated, it could lead to post-harvest Fuzi rot, and could be re-isolated from symptomatic Fuzi and had the same cultural, physiological, and biochemical characteristics as that inoculated.

### Identification and Characterization of the Isolate X2

The Fuzi inoculated with isolate X2 also showed symptoms that were identical to those observed in rotten post-harvest Fuzi. Following inoculation with X2, red lesions appeared that were sunken, and the whole Fuzi was brown and soft (Figure 1G). The individual colonies were smooth, round, and red (or white) in color, and the individual cells were straight, round-ended rods with dimensions of 0.6–0.9 × 1.0–1.5 μm (Figures 1H,I). This bacterium could grow at temperatures of 37 and 40°C, but not 5°C. It could also grow in the presence of 7% (w/v) NaCl, but not 10% NaCl. It was able to assimilate L-malate, glycerol, D-galactose, gentiobiose, D-glucose, lactose, D-mannose, D-melibiose, sucrose, D-trehalose, L-arabinose, D-galacturonate, L-glutamate, D-xylose, citrate, glucuronate, and L-proline but could not assimilate D-cellobiose, erythritol, D-raffinose, D-melezitose, L-sorbose, L-rhamnose, D-turanose, or nitrate. The methyl red test was negative.

The 16S rRNA gene of X2 was 1,442 bp in length (MW652658.1) and showed 99.8% identity with the 16S rRNA genes from several other strains of *S. marcescens* identified by an NCBI BLAST query (MT434357.1 and MN519524.1) (Figure 2B). Moreover, the housekeeping gene *pigC* of X2 (MW691199) showed 99.8% identity with the *S. marcescens* strains (CP0313161.1 and AP019009.1). The MALDI-TOF MS results indicated that X2 was *S. marcescens* (confidence > 99.0%) (Supplementary Figure 2B and Supplementary Table 2). Therefore, based on the morphological and physiological observations, the phylogenetic analysis, and the MALDI-TOF MS results, isolate X2 was classified as *S. marcescens*. Moreover, after X2 was reinoculated, it could also lead to post-harvest Fuzi rot, and could be re-isolated from symptomatic Fuzi and had the same cultural, physiological, and biochemical characteristics as that inoculated.

### Establishment of the LAMP-Based Methods

**FIGURE 3:**
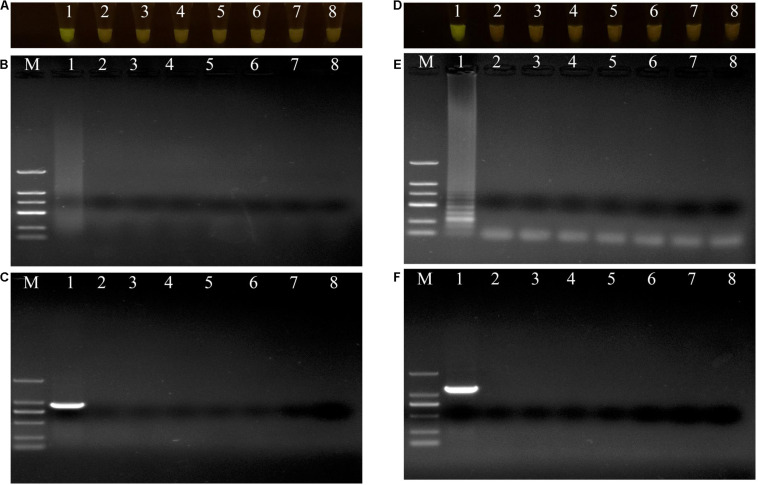
Evaluations of specificity of loop-mediated isothermal amplification (LAMP) and polymerase chain reaction (PCR) assay. **(A–C)** Evaluation of specificity of LAMP and PCR assay for *Pseudomonas aeruginosa* X1 and the other close bacterial strains. Lane M: DNA marker DL2000; number 1–8: *Pseudomonas aeruginosa* X1, *Pseudomonas syringae* DC3000, *Pseudomonas putida* X13, *Pseudomonas psychrotolerans* 1.15631, *Pseudomonas nitroreducens* 1.1796, *Pseudomonas. toyotomiensis* 1.532, *Pseudomonas fluorescens* 1.4528, ddH_2_O. **(D–F)** Evaluation of specificity of LAMP and PCR assay for *Serratia marcescens* X2 and the other close bacterial strains. Lane M: DNA marker DL2000; number 1–8: *Serratia marcescens* X2, *Serratia liquefaciens* BNCC 186068, *Serratia odorifera* Bio-67784, *Serratia plymuthica* 1.996, *Serratia fonticola* 1.995, *Serratia nematodiphla* 1.6853, *Serratia rubidaea* 1.10839, ddH_2_O. **(A**,**D)** LAMP products visualized with gelgreen. Under daylight, positive reactions are shown by bright green color, and negative reactions are dark red color. **(B**,**E)** Determining the visual LAMP products by agarose gel electrophoresis. **(C**,**F)** Determining the PCR products by agarose gel electrophoresis.

The specificities of the housekeeping genes and the LAMP primers were tested using genomic DNAs obtained from the two isolates X1 and X2, and the other closely related bacterial strains (Supplementary Table 1). Through conventional PCR, an 864-bp-long band from the housekeeping gene *gyrB* and a 1138-bp-long band from the housekeeping gene *pigC* were amplified in *P. aeruginosa* X1 (Figure 3C) and *S. marcescens* X2 (Figure 3F), respectively, but not in the other strains or the control (ddH_2_O). Furthermore, bright green reaction products were only observed in the samples containing DNAs of *P. aeruginosa* X1 (Figure 3A) and *S. marcescens* X2 (Figure 3D). while dark red reaction products were observed for the other bacterial samples and the control. Finally, the electrophoresis results showed that the specific trapezoidal bands were only amplified in the samples of *P. aeruginosa* X1 (Figure 3B) and *S. marcescens* X2 (Figure 3E), and not in the other bacterial samples or the control. All of these results confirmed that the housekeeping gene *gyrB*, and *pigC*, and their target regions could discriminate the two bacteria from other closely related species.

**FIGURE 4:**
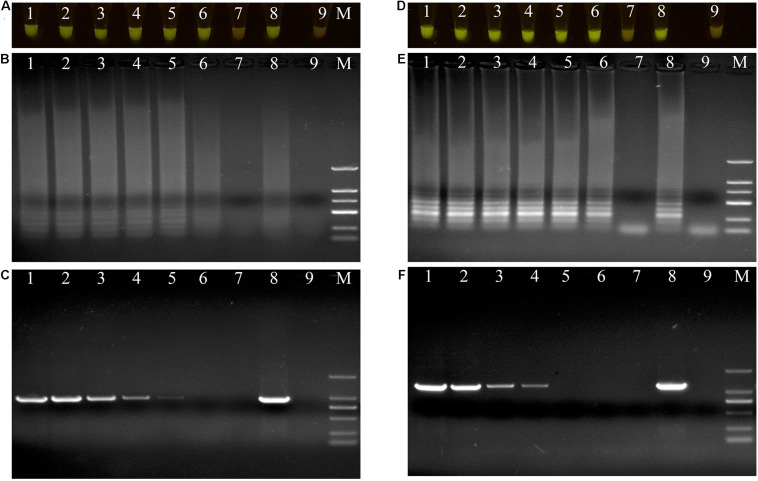
Evaluations of sensitivity of loop-mediated isothermal amplification (LAMP) and polymerase chain reaction (PCR) assay. **(A–C)** Evaluation of sensitivity of LAMP and PCR assay for *Pseudomonas aeruginosa* X1. Lane M: DNA marker DL2000; number1–7: DNA at concentrations of 20 ng/μL, 2 ng/μL, 200 pg/μL, 20 pg/μL, 2 pg/μL, 200 fg/μL, and 20 fg/μL. number 8: positive control (*Pseudomonas aeruginosa* X1). number 9: ddH_2_O. **(D–F)** Evaluation of sensitivity of LAMP and PCR assay for *Serratia marcescens* X2. Lane M: DNA marker DL2000; number 1–7: DNA at concentrations of 20 ng/μL, 2 ng/μL, 200 pg/μL, 20 pg/μL, 2 pg/μL, 200 fg/μL, and 20 fg/μL. number 8: positive control (*Serratia marcescens* X2). number 9: ddH_2_O. **(A**,**D)** LAMP products visualized with gelgreen. Under daylight, positive reactions are shown by bright green color, and negative reactions are dark red color. **(B**,**E)** Determining the visual LAMP products by agarose gel electrophoresis. **(C**,**F)** Determining the PCR products by agarose gel electrophoresis.

The DNAs of the two bacterial isolates were serially diluted 10-fold from 20 ng/μL to 20 fg/μL, and used as DNA templates. After amplification, the reaction mixtures for *P. aeruginosa* X1 were bright green when the DNA concentration was more than 20 fg/μL (Figure 4A). The electrophoresis results further showed that when the DNA concentration was more than 20 fg/μL, there was a specific trapezoid strip, whereas when the DNA concentration was 20 fg/μL, no specific trapezoid strip was observed, which was consistent with the fluorescence detection results (Figure 4B). The sensitivity of PCR was 2 pg/μL for both *P. aeruginosa* X1 (Figure 4C) and *S. marcescens* X2 while the sensitivity of LAMP was 10- and 100-fold higher than this for each species, respectively (Figures 4D–F).

### Application of LAMP and Comparison With Culture-Based Assays

**FIGURE 5:**
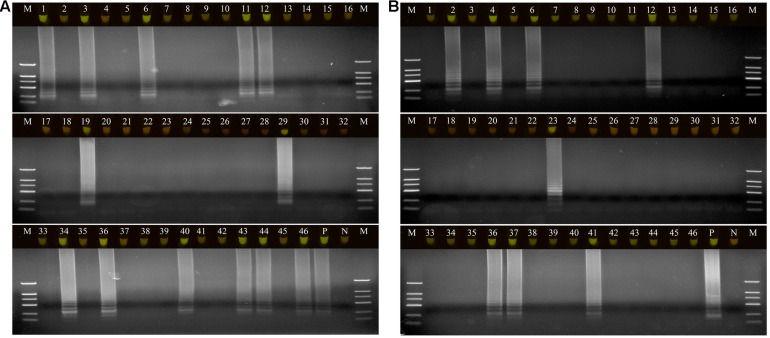
The detection results of *Pseudomonas aeruginosa* by loop-mediated isothermal amplification (LAMP) in Fuzi and rhizosphere soil samples. **(A)** The detection results of *Pseudomonas aeruginosa* by LAMP in Fuzi samples. Lanes 1–46: different Fuzi samples. **(B)** The detection results of *Pseudomonas aeruginosa* by LAMP in Fuzi rhizosphere soil samples. Lanes 1–46: different Fuzi rhizosphere soil samples. **(Up)** LAMP products visualized with gelgreen. Under daylight, positive reactions are shown by bright green color, and negative reactions are dark red color. **(Down)** LAMP products determined by agarose gel electrophoresis. Lane M: DNA marker DL2000. Lane P: positive control (*Pseudomonas aeruginosa* X1). Lane N: negative control (ddH_2_O).

**FIGURE 6:**
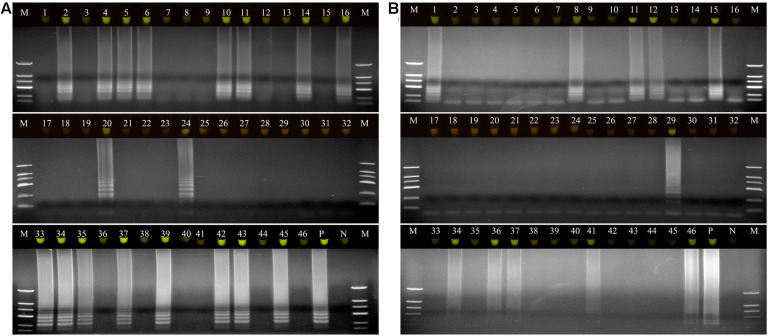
The detection results of *Serratia marcescens* by loop-mediated isothermal amplification (LAMP) in Fuzi and rhizosphere soil samples. **(A)** The detection results of *Serratia marcescens* by LAMP in Fuzi samples. Lanes 1–46: different Fuzi samples. **(B)** The detection results of *Serratia marcescens* by LAMP in Fuzi rhizosphere soil samples. Lanes 1–46: different Fuzi rhizosphere soil samples. **(Up)** LAMP products visualized with gelgreen. Under daylight, positive reactions are shown by bright green color, and negative reactions are dark red color. **(Down)** LAMP products determined by agarose gel electrophoresis. Lane M: DNA marker DL2000. Lane P: positive control (*Serratia marcescens* X2). Lane N: negative control (ddH_2_O).

A total of 46 naturally occurring Fuzi and their associated rhizosphere soil samples were evaluated using the LAMP assays to detect *P. aeruginosa* and *S. marcescens*. Among these, 13 of the Fuzi (Figure 5A) and 8 of the soil (Figure 5B) samples tested positive for *P. aeruginosa* in the LAMP assay. Similarly, 18 of the Fuzi (Figure 6A) and 11 of the soil (Figure 6B) samples tested positive in the *S. marcescens* assay. Moreover, *P. aeruginosa* could be isolated from four of Fuzi and three of the soil samples, and *S. marcescens* could be isolated from six of Fuzi and three of the soil samples (Supplementary Table 3).

## Discussion

In China’s main planting regions of *A. carmichaelii*, post-harvest Fuzi rot can spread quickly, which can easily lead to devastation of the crop. Therefore, it is important that the main pathogens associated with post-harvest Fuzi rot are identified and suitable detection methods for them are developed to help establish economic methods for preventing such losses. In this study, two new pathogenic bacteria, *P. aeruginosa* X1 and *S. marcescens* X2, were isolated from rotten Fuzi and rapid and specific detection methods were established for them.

Both *P. aeruginosa* and *S. marcescens* can be found in many natural environments, including the atmosphere, the soil, plants, water, and hospitals (Besler and Little, 2017; Haddoudi et al., 2017; Chegini et al., 2020; Tan et al., 2020). Different strains of these species show diverse characteristics, with various strains having been proposed as pathogen/insect biocontrol agents, plant growth promotion bacteria (PGPBs) (Singh and Jha, 2016; Haddoudi et al., 2017; Kumawat et al., 2019; Al-Ghafri et al., 2020; Chandra et al., 2020; Turhan et al., 2020), and opportunistic human pathogens (Besler and Little, 2017; Tan et al., 2020). In particular, some *P. aeruginosa* strains show high intrinsic resistances to a range of antibiotics, resulting in significant morbidity and mortality rates (Chegini et al., 2020). In terms of plant diseases, several strains of *P. aeruginosa* can cause root rot in ginseng (*Panax ginseng*) (Gao et al., 2014), internal brown rot in stored onion (*Allium cepa*) bulbs (Abd-Alla et al., 2011), fruit rot in round melon (*Praecitrullus fistulosus*) (Mondal et al., 2012), and leaf disease in soybean (*Glycine max*) and lettuce (*Lactuca sativa*) (Plasencia-Márquez et al., 2017). Moreover, some strains of *S. marcescens* can induce lesions and necrosis in tobacco (*Nicotiana tabacum*) leaves, wilt virus in carrot (*Daucus carota*), yellow vine disease in sunflowers (*Helianthus* spp.), leaf chlorosis and necrotic spots in squash (*Cucurbita pepo* var. *styriaca*), leaf spot disease in industrial hemp (*Cannabis sativa*), soft rot in ginger (*Zingiber officinale*) rhizomes and many other diseases (Huang et al., 2020; Schappe et al., 2020). In this study, strains X1 and X2 were isolated from rotten post-harvest Fuzi and were identified as *P. aeruginosa* and *S. marcescens*, respectively, based on their morphological, physiological, and biochemical characteristics, housekeeping gene homologies, and MALDI-TOF MS results. The pathogenicities of these isolates were confirmed by fulfilling Koch’s postulates demonstrating they were post-harvest Fuzi rot pathogens. To our knowledge, this is the first report of *P. aeruginosa* and *S. marcescens* causing post-harvest Fuzi rot.

Although strains of both *P. aeruginosa* and *S. marcescens* strains can be PGPBs or pathogens of humans, animals, or plants, these species are not typical plant pathogens as they do not ordinarily cause visible damage to plants unless they are subjected to moderate to high temperatures and high moisture conditions. In this study, *P. aeruginosa* X1 and *S. marcescens* X2 were confirmed as post-harvest Fuzi rot pathogens. Other research has shown that the capacity of clinical isolates of *P. aeruginosa* to induce rot in vegetables was indistinguishable from that of agricultural isolates (Schroth et al., 2018). For instance, two clinical isolates from burn patients, *P. aeruginosa* PA13 and PA14, exhibited significant virulence in causing rot in all of the tested plants, especially on cucumber (*Cucumis sativus*), lettuce, potato (*Solanum tuberosum*), and tomato (*Solanum lycopersicum*) (Schroth et al., 2018). Moreover, *Serratia marcescens* YC16 isolated from rotten ginger has the potential to infect mammalian cells (Huang et al., 2020).

To date, detection methods for *P. aeruginosa* and *S. marcescens* have mainly focused on the human pathogen candidates. A number of methods have been used as diagnostic tools in clinical settings to identify *P. aeruginosa* in patient samples, such as culture-based assay, the high-throughput immunochemical method, the electrochemical detection method, and qRT-qPCR (Costaglioli et al., 2014; Alatraktchi et al., 2020; Montagut et al., 2020). However, the time and materials required for culture-based assays limit the number of samples that can be screened (Joyner et al., 2014), while the other methods are relatively difficult to operate, require expert technicians, and cannot provide real-time detection, making them less suitable for on-field testing and early warning systems (Fang and Ramasamy, 2015). Cost effective techniques based on LAMP have now emerged as substitutes for PCR because of their simplicity (only a heating block or water bath that is capable of maintaining a constant temperature of 60–65°C is needed), rapidity, specificity, and sensitivity. For instance, Kim et al. (2016) developed a LAMP method to rapidly detect nosocomial carbapenem-resistant *P. aeruginosa* strains, using the β-lactamase genes *bla*_VIM__–__2_ and *bla*_IMP__–__1_ as targets, while Takano et al. (2019) established a novel LAMP method for assaying guiana extended-spectrum (GES) β-lactamase-producing *P. aeruginosa* strains in hospitalized patients through the detection of the gene *bla*_GES_. In addition, Manajit et al. (2018) developed a uracil-DNA-glycosylase-supplemented loop-mediated isothermal amplification coupled with nanogold probe (UDG-LAMP-AuNP) method to specifically detect *P. aeruginosa* based on the extracytoplasmic function gene (*ecfX*). MALDI-TOF MS, qRT-PCR, repetitive element palindromic PCR, and next-generation sequencing methods have also been used to detect *S. marcescens* and identify potential environmental sources of infections (Iwaya et al., 2005; Joyner et al., 2014; Rödel et al., 2019; Yeo et al., 2019).

In the present study, two LAMP methods were successfully developed that targeted the genes *gyrB* of *P. aeruginosa*, and *pigC* of *S. marcescens*. The target genes and the LAMP methods were specific to these two pathogens, and LAMP methods had approximately a 10-fold higher sensitivity than conventional PCR. The established LAMP methods were also used to detect *P. aeruginosa* and *S. marcescens* in 92 samples of unknown etiology, which included healthy and rotten Fuzi and their associated rhizospheric soil samples. The results showed that the two bacteria could be detected in several of the healthy Fuzi and their rhizospheric soil samples, indicating that they had colonized and survived in Fuzi and the soil but had not induced a rotten appearance. In the main planting regions of *A. carmichaelii*, Fuzi is harvested and directly used in culture without any washing or sterilizing procedures. Thus, it is likely that the Fuzi and/or soil act as “carriers” of *P. aeruginosa* or *S. marcescens* likely increasing the risk of *A. carmichaelii* infection with the two pathogens. In the growing season, live *A. carmichaelii* cultivars that are colonized by the two bacteria would show no disease symptoms due to the host defense mechanisms. However, the presence of these bacteria would result in drastic losses of the post-harvest Fuzi under high temperature and high moisture conditions. Other procedures, such as storage and transit, also represent potential sources of contamination by the two pathogens. Therefore, these agricultural practices should be examined as potential sources of *P. aeruginosa* and *S. marcescens*.

## Conclusion

In conclusion, *P. aeruginosa* X1 and *S. marcescens* X2 were isolated, identified, and associated with post-harvest Fuzi rot and novel LAMP methods were developed for the detection of the two pathogens. The developed methods are rapid, convenient, efficient, specific, and sensitive, being able to be accomplished within 1 h at 65°C and not requiring any complex or expensive instruments. Thus, they represent very innovative, convenient, cheap, and rapid diagnostic tools. These methods are expected to provide point-of-care testing for the two pathogens under different Fuzi planting procedures, which will significantly contribute to the control and prevention of the Fuzi rot.

## Data Availability Statement

The datasets presented in this study can be found in online repositories. The names of the repository/repositories and accession number(s) can be found below: https://www.ncbi.nlm.nih.gov/, MW652657.1, MW652658.1, MW691198, and MW691199.

## Author Contributions

MF, XZ, and BC: formal analysis and writing. GZ: investigation. ML and LC: review, editing, and project administration. All authors have read and agreed to the published version of the manuscript.

## Conflict of Interest

The authors declare that the research was conducted in the absence of any commercial or financial relationships that could be construed as a potential conflict of interest.

## Publisher’s Note

All claims expressed in this article are solely those of the authors and do not necessarily represent those of their affiliated organizations, or those of the publisher, the editors and the reviewers. Any product that may be evaluated in this article, or claim that may be made by its manufacturer, is not guaranteed or endorsed by the publisher.

## Footnotes

1 NCBI, www.ncbi.nlm.nih.gov.

2
https://primerexplorer.jp/
