# Supplementary material for: Characteristics of Isolates of Pseudomonas aeruginosa and Serratia marcescens Associated With Post-harvest Fuzi (Aconitum carmichaelii) Rot and Their Novel Loop-Mediated Isothermal Amplification Detection Methods
Source: Front Microbiol. 2021 Aug 20;12:705329. doi: 10.3389/fmicb.2021.705329 (PMC8417746; doi:10.3389/fmicb.2021.705329)
Supplement: Supplementary file 1 [file Data_Sheet_1.docx]

Supplementary Material

## Supplementary Figures


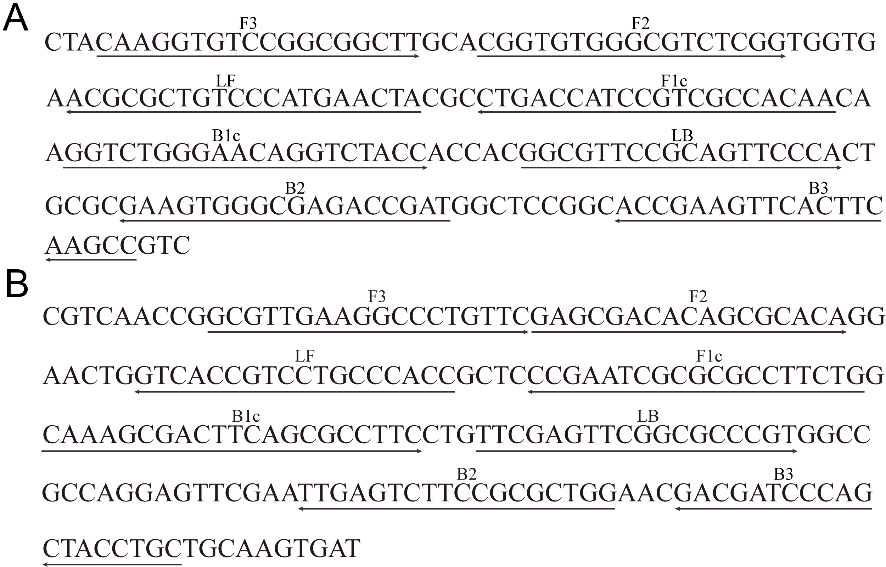


Supplementary Figure 1. Primer design of loop-mediated isothermal amplification (LAMP)

Note: A.LAMP primers of *Pseudomonas aeruginosa*; B. LAMP primers of  [*Serratia marcescens*](https://blast.ncbi.nlm.nih.gov/Blast.cgi#alnHdr_1216679061)*.* DNA sequences used for primer design are underlined.

LF: loop forward primer; LB: loop backward primer; FIP: forward inner primer; BIP: backward inner primer; F3: forward primer; B3: backward primer.


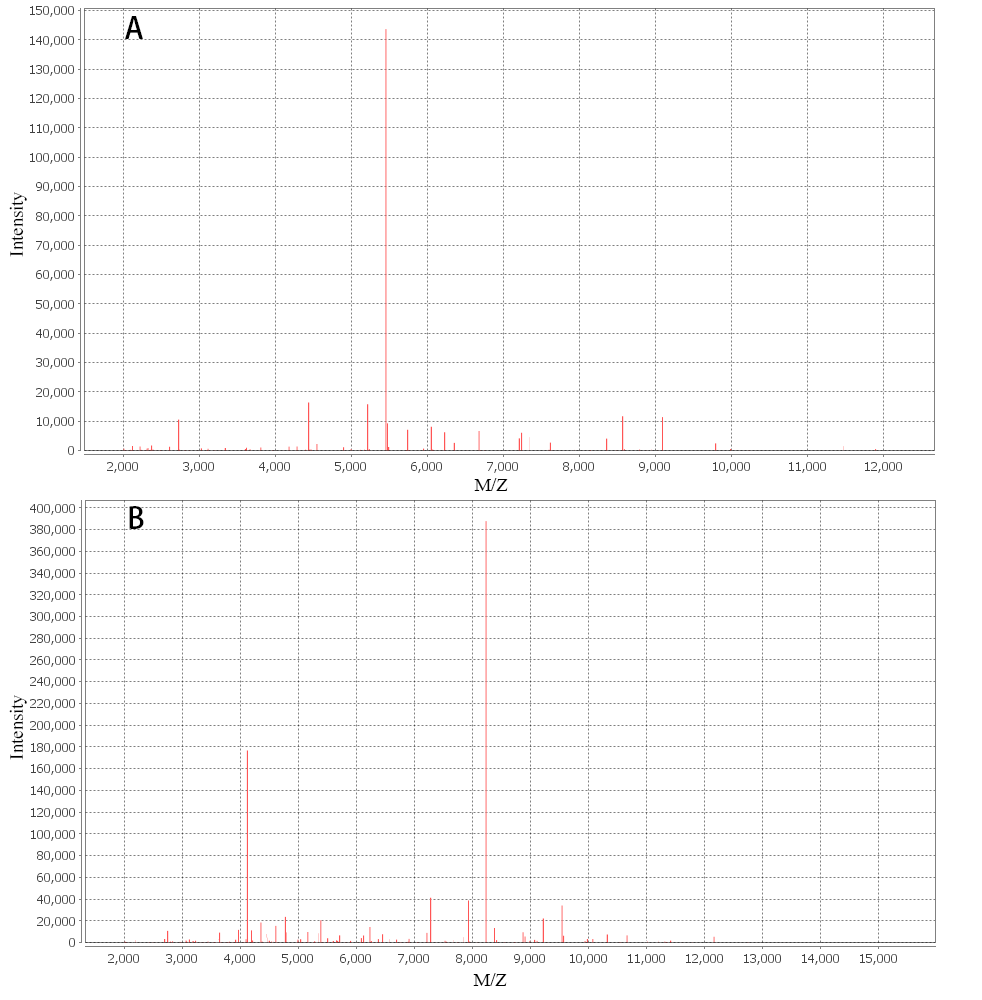


Supplementary Figure 2. Identification results by MALDI-TOF MS

Note: **A.** Intensity of *Pseudomonas aeruginosa*; **B.** Intensity of  [*Serratia marcescens*](https://blast.ncbi.nlm.nih.gov/Blast.cgi#alnHdr_1216679061)*.*

## Supplementary table

Supplementary Table 1. Bacterial species and strains used in this study to check the specificity of LAMP primers

| Species | Isolate | Source |
| --- | --- | --- |
| *Pseudomonas aeruginosa* | X1 | National Engineering Laboratory for Resource Development of Endangered Crude Drugs in Northwest China (NELRD) |
| *Pseudomonas syringae* | DC3000 | NELRD |
| *Pseudomonas putida* | X13 | NELRD |
| *Pseudomonas psychrotolerans* | 1.15631 | China General Microbiological Culture Collection Center (CGMCCC) |
| *Pseudomonas nitroreducens* | 1.1796 | CGMCCC |
| *Pseudomonas toyotomiensis* | 1.532 | Institute of microbiology, Guangdong academy of sciences |
| *Pseudomonas fluorescens* | 1.4528 | CGMCCC |
| *Serratia marcescens* | X2 | NELRD |
| *Serratia liquefaciens* | BNCC 186068 | Be Na Culture Collection(BNCC) |
| *Serratia odorifera* | Bio-67784 | Biobw |
| *Serratia plymuthica* | 1.996 | Institute of microbiology, Guangdong academy of sciences |
| *Serratia fonticola* | 1.995 | Institute of microbiology, Guangdong academy of sciences |
| *Serratia nematodiphila* | 1.6853 | CGMCCC |
| *Serratia rubidaea* | 1.10839 | CGMCCC |

Supplementary Table 2. The identification results of *Pseudomonas aeruginosa* and *Serratia marcescens* with VITEK MS system

| Reference Identification | VITEK MS Result | Similarity |
| --- | --- | --- |
| X1 | *Pseudomonas aeruginosa* | 99.9% |
| X2 | *Serratia marcescens* | 99.9% |

Supplementary Table 3. Isolation of *Pseudomonas aeruginosa* and  [*Serratia marcescens*](https://blast.ncbi.nlm.nih.gov/Blast.cgi#alnHdr_1216679061) in Fuzi and their rhizosphere soil samples

| Number | Type | [Appearance](https://www.baidu.com/link?url=Ph3YZlqxSrMVPgkH8qaNj5ZgBIukXYMuVDpQ4ZiBEYhlTk-cIMIPOhyDcSV-TcHh_MqW7tNETUru1zXy5rAg_PR9WGrfFVfF6f1TX4KRyk3&wd=&eqid=c6d2ce99001c7de400000006603a55cb) | PA^a^ | SM^b^ | Number | Type | PA^a^ | SM^b^ | Location |
| --- | --- | --- | --- | --- | --- | --- | --- | --- | --- |
| 1 | Fuzi | Rot | - | - | 1# | soil | - | + | HS^c^ |
| 2 | Fuzi | Rot | - | - | 2# | soil | - | - | HS |
| 3 | Fuzi | Healthy | - | - | 3# | soil | - | - | HS |
| 4 | Fuzi | Rot | - | - | 4# | soil | - | - | HS |
| 5 | Fuzi | Rot | - | - | 5# | soil | - | - | HS |
| 6 | Fuzi | Rot | - | - | 6# | soil | - | - | HS |
| 7 | Fuzi | Healthy | - | - | 7# | soil | - | - | HS |
| 8 | Fuzi | Rot | - | - | 8# | soil | - | + | HS |
| 9 | Fuzi | Healthy | - | - | 9# | soil | - | - | HS |
| 10 | Fuzi | Rot | - | - | 10# | soil | - | - | HS |
| 11 | Fuzi | Rot | - | + | 11# | soil | - | + | HS |
| 12 | Fuzi | Rot | + | - | 12# | soil | + | - | HS |
| 13 | Fuzi | Healthy | - | - | 13# | soil | - | - | HS |
| 14 | Fuzi | Rot | - | - | 14# | soil | - | - | HS |
| 15 | Fuzi | Healthy | - | - | 15# | soil | - | - | HS |
| 16 | Fuzi | Rot | - | - | 16# | soil | - | - | HS |
| 17 | Fuzi | Healthy | - | - | 17# | soil | - | - | HS |
| 18 | Fuzi | Healthy | - | - | 18# | soil | - | - | HS |
| 19 | Fuzi | Rot | + | - | 19# | soil | - | - | HS |
| 20 | Fuzi | Healthy | - | - | 20# | soil | - | - | HS |
| 21 | Fuzi | Rot | - | - | 21# | soil | - | - | LY^d^ |
| 22 | Fuzi | Rot | - | - | 22# | soil | - | - | LY |
| 23 | Fuzi | Rot | - | - | 23# | soil | - | - | LY |
| 24 | Fuzi | Rot | - | - | 24# | soil | - | - | LY |
| 25 | Fuzi | Rot | - | - | 25# | soil | - | - | LY |
| 26 | Fuzi | Healthy | - | - | 26# | soil | - | - | LY |
| 27 | Fuzi | Healthy | - | - | 27# | soil | - | - | LY |
| 28 | Fuzi | Healthy | - | - | 28# | soil | - | - | LY |
| 29 | Fuzi | Healthy | - | - | 29# | soil | - | - | LY |
| 30 | Fuzi | Healthy | - | - | 30# | soil | - | - | MS^e^ |
| 31 | Fuzi | Healthy | - | - | 31# | soil | - | - | MS |
| 32 | Fuzi | Healthy | - | - | 32# | soil | - | - | MS |
| 33 | Fuzi | rot | - | + | 33# | soil | - | - | MS |
| 34 | Fuzi | Rot | + | - | 34# | soil | - | - | MS |
| 35 | Fuzi | Rot | - | - | 35# | soil | - | - | MS |
| 36 | Fuzi | Rot | + | - | 36# | soil | + | - | MS |
| 37 | Fuzi | Rot | - | + | 37# | soil | - | - | MS |
| 38 | Fuzi | Healthy | - | - | 38# | soil | - | - | MS |
| 39 | Fuzi | Rot | - | + | 39# | soil | - | - | MS |
| 40 | Fuzi | Rot | - | - | 40# | soil | - | - | MS |
| 41 | Fuzi | Rot | - | - | 41# | soil | + | - | MS |
| 42 | Fuzi | Healthy | - | - | 42# | soil | - | - | MS |
| 43 | Fuzi | Rot | - | + | 43# | soil | - | - | MS |
| 44 | Fuzi | Rot | - | - | 44# | soil | - | - | MS |
| 45 | Fuzi | Rot | - | + | 45# | soil | - | - | MS |
| 46 | Fuzi | Rot | - | - | 46# | soil | - | - | MS |

Note:

**a:** *Pseudomonas aeruginosa*;  **b:**  [*Serratia marcescens*](https://blast.ncbi.nlm.nih.gov/Blast.cgi#alnHdr_1216679061); **c:** Hanzhong, Shaanxi Province, China; **d:** Lijiang, Yunnan Province, China. **e:** Mianyang, Sichuan Province, China.

+: The bacterium could be isolated in the sample.

-: The bacterium could not be isolated in the sample.
